# Development of a New Risk Score for Stratification of Women with Gestational Diabetes Mellitus at High Risk of Persisting Postpartum Glucose Intolerance Using Routinely Assessed Parameters

**DOI:** 10.3390/life11060464

**Published:** 2021-05-23

**Authors:** Vendula Bartáková, Beáta Barátová, Lukáš Pácal, Veronika Ťápalová, Silvie Šebestová, Petr Janků, Kateřina Kaňková

**Affiliations:** 1 Department of Pathophysiology, Faculty of Medicine, Masaryk University, 625 00 Brno, Czech Republic; bea.baratova@gmail.com (B.B.); paci@med.muni.cz (L.P.); kankov@med.muni.cz (K.K.); 2 Department of Pediatrics, University Children Hospital, Černopolní 9, 613 00 Brno, Czech Republic; 3 Department of Obstetrics and Gynaecology, University Hospital Brno, 625 00 Brno, Czech Republic; tapalova.veronika@fnbrno.cz (V.Ť.); janku.petr@fnbrno.cz (P.J.); 4 Institute of Biostatistics and Analyses, Faculty of Medicine, Masaryk University, 625 00 Brno, Czech Republic; amice@seznam.cz; 5 Department of Nursing and Midwifery, Masaryk University, 625 00 Brno, Czech Republic

**Keywords:** gestational diabetes mellitus, persisting glucose intolerance postpartum, risk score, peripartal outcomes

## Abstract

The aims of the study were (i) to find predictive factors for early postpartum conversion of gestational diabetes mellitus (GDM) into persisting glucose intolerance (PGI), (ii) to evaluate potential differences in adverse perinatal outcomes in GDM women with and without early postpartum PGI and, finally, (iii) to establish a risk score to predict postpartum PGI. A cross-sectional study comprised 244 GDM patients with known age, parity, positive family history of diabetes, pre-gestational BMI, comorbidities, smoking history, results of mid-trimester oral glucose tolerance test, HbA1c, obstetric complications, neonatal outcomes and mode of delivery. A risk score was calculated using parameters with highest odds ratios in a statistic scoring model. Significant differences between women with and without PGI postpartum were ascertained for mid-trimester fasting plasma glucose (*p* < 0.001), HbA1c above 42 mmol/mol (*p* = 0.035), prevalence of obesity (*p* = 0.007), hypothyroidism, family history of diabetes and smoking. We also observed higher incidence of prolonged and complicated delivery in PGI group (*p* = 0.04 and 0.007, respectively). In conclusion, this study identified several parameters with predictive potential for early PGI and also adverse peripartal outcomes. We established a simple risk-stratification score for PGI prediction applicable for GDM affected women prior their leaving maternity ward. Yet, given a relatively small sample size as a main limitation of this study, the proposed score should be validated in the larger cohort.

## 1. Introduction

Gestational diabetes mellitus (GDM) is defined as any degree of glucose intolerance first diagnosed in pregnant women (most often during the period of 24th to 28th week of gestation by a compulsory oral glucose tolerance test (oGTT)), that usually disappears after delivery [1]. As the prevalence of GDM is rising worldwide [2]—due to the change in the lifestyle (mainly overweight/obesity and postponed motherhood) and partly due to the change of diagnostic criteria—there is a growing interest in this particular complication of pregnancy. In 2017, 16.2% of pregnant women had been reported to have some form of hyperglycaemia, 86.4% of those were due to GDM. This means that one in seven births is affected by GDM, which therefore represents a threat to the health of both mother and child [3].

Several published papers showed that women with GDM are at higher risk of not only having complications during delivery, but also of maintaining abnormalities in glucose metabolism after delivery. They are seven times more likely to develop prediabetes or type 2 diabetes mellitus (T2DM) in future life [4,5]. At the same time, GDM represents risk for the offspring, who is more likely to have macrosomia, hypoglycaemia, hyperbilirubinemia or respiratory distress syndrome after delivery. Children of mothers with GDM are also more prone to suffer from childhood obesity and cardiovascular diseases (CVD) or to develop T2DM more frequently in subsequent life [6].

The etiopathogenesis of GDM is still incompletely understood. While an increased insulin sensitivity is typical for the first phase of physiological gestation, an insulin resistance develops in mid to late gestation as a consequence of rising concentration of maternal (such as oestrogen, progesterone and leptin) and placental hormones (cortisol, prolactin, human placental lactogen and growth hormone) counter-acting insulin action [7]. Healthy women are able to raise the insulin production and compensate for the defect, whereas women with a latent defect of insulin secretion are not. This inability later manifests as GDM. Insulin resistance may develop earlier (14–16th week of gestation) in women with pre-existing impairment of glucose metabolism [8]. Higher body mass index (BMI) before pregnancy, older maternal age, smoking and positive family history of diabetes (DM) are all considered as risk factors for developing GDM [9].

The risk of persisting impaired glucose tolerance (PGI) after delivery is not the same for every woman [10]. This could reflect likely etiopathogenetic heterogeneity of the disease (e.g., various contribution of genetic, environmental and other factors). By identifying women with GDM who are at higher risk of PGI early postpartum, we can implement earlier intervention (by personalised education and lifestyle modifications) and ensure more intense postpartum follow-up.

The aims of our pilot study were (i) to find eventual predictive factors for early postpartum conversion of GDM into PGI using anthropometric, biochemical and clinical data from the second trimester focusing on potential differences between women with normal BMI, overweight and obesity, (ii) to evaluate potential differences in adverse perinatal outcomes in GDM women with and without PGI early postpartum and (iii) to establish a simple risk score to predict postpartum PGI using routinely assessed parameters which can be used for stratification of GDM women before their leaving the maternity ward after delivery.

## 2. Materials and Methods 

### 2.1. Subjects

A cross-sectional observational study included a total of 244 participants having GDM (all Caucasian of Czech nationality from South Moravian Region, Czech Republic), who were followed between 2011–2013 and who at the same time underwent repeated oGTT test up to 1 year after delivery. Parameters including glucose values during postpartum oGTT and those related to labour and offspring were extracted during 2015–2016 by investigators from hospital electronic health records. Since data on breastfeeding (yes/no and its eventual duration) were not available in electronic health records, this parameter was not considered in subsequent analyses. Exclusion criteria were: established diabetes mellitus type 1 or 2 before pregnancy (diagnosed according to recent WHO criteria [11]), non-Caucasian origin and multiple pregnancies. 

### 2.2. Methods

All subjects underwent routine mid-gestational GDM screening by oral glucose tolerance test (oGTT) with 75 g of glucose between 24–28th week of pregnancy (mid trimester). GDM was diagnosed according to the old WHO criteria: fasting plasma glucose (FPG) ≥ 5.6 mmol/L, 1-h post-load glucose ≥ 8.9 mmol/L and 2-h post-load glucose ≥ 7.7 mmol/L (reaching any of the three cut-off values qualified for the GDM diagnosis). All participants were followed from the time of GDM diagnosis until the delivery at the Diabetes Centre of the Faculty Hospital Brno. Treatment of GDM included diet in all cases, whereby 37.3% of GDM cases required insulin therapy. Postpartum diagnosis of diabetes/prediabetes was based on the WHO criteria for non-pregnant subjects: FPG ≥ 7 mmol/L alone or 2-h post-load glucose ≥ 11.1 mmol/L for diabetes mellitus, FPG 5.6–6.9 mmol/L or 2-h post-load glucose 7.8–11.0 mmol/L for prediabetes. In case of positive postpartum test for manifest diabetes urinary ketone bodies, C-peptide and selected antibodies (anti-glutamic acid decarboxylase, anti-tyrosine phosphatase–2 and insulin autoantibodies) were measured to identify eventual type 1 diabetes (T1DM). Majority of GDM women (95%) participated in the repeated oGTT immediately after puerperium (6 weeks after delivery) as scheduled by in, remaining 5% booked later term any time up to the 12 months post-delivery. 

Peripartal and biochemical parameters were extracted by investigators from hospital electronic health records, other data were obtained from questionnaires (available in authors) developed by investigators that were completed by study subjects and their diabetologist at the time of GDM diagnosis (second trimester of gravidity, after oGTT test). All data were transferred to the electronic database (available in authors). Following parameters were considered for analysis: age at the time of GDM diagnosis, parity, family history of diabetes, pre-gestational BMI, personal history of selected comorbidities known to have higher prevalence in GDM (hypothyroidism, anaemia, thrombophilia, allergy, hypertension or preeclampsia, polycystic ovary syndrome—all diseases diagnosed according to recent WHO criteria), smoking, selected biochemical parameters at the time of GDM diagnosis (glycaemia during oGTT test in the mid-trimester, glycated haemoglobin (HbA1c)), delivery-related parameters—such as term of delivery (delivery before the 38th week of gestation was considered as preterm in accordance with the current national nomenclature), length of delivery (prolonged delivery above 480 min, all three stages of labour are counted) and necessity for its induction, instrumental delivery or caesarean section and postpartum complications (such as manual extraction of placenta or hypotonia uteri) and, finally, selected neonatal parameters (Apgar score, pH of cord blood, base excess (BE) and child birth weight). Additionally, area under curve (AUC, mmol/L/h) was calculated from a 3-point oGTT in mid-trimester using the trapezoid rule [12].

Study was approved by the Ethical Committee of Faculty of Medicine, Masaryk University, Brno, Czech Republic, and was conducted in accordance with Helsinki declaration (approval number 22/2010, date of approval 16 September 2010). Each participant provided informed consent prior being included in the study.

### 2.3. Statistical Analysis

Data are expressed as medians and interquartile ranges (IQR) or percentage for between-group comparisons. Shapiro–Wilk normality test was used for testing of normal distribution. Nonparametric tests were used for comparison between and within the groups (Mann–Whitney and Wilcoxon tests, respectively). Chi-square test was used for contingency tables. Software Statistica (StatSoft, Tulsa, OK, USA) was used for all analyses. *p* < 0.05 was considered statistically significant.

Univariate and multivariate logistic models were constructed to determine an eventual statistically significant effect of any relevant variable and receiver operating characteristic (ROC) analysis was applied to test the final models. Areas under the ROC curve (AUC/ROC) were compared by Delong paired test [13]. Optimal cut-offs of selected glycaemic indices for prediction of postpartum GDM conversion into permanent glucose abnormality within 12 month were selected by the highest Youden indices [14], i.e., single statistic capturing diagnostic test performance (J = sensitivity + specificity − 1) with value ranging from 0 to 1 (a zero value for the test giving the same proportion of positive results for groups with and without the disease and a value of 1 for no false positives or negatives). 

For risk score development, univariate and multivariate logistic regression with backward stepwise prediction algorithm was adopted for the analysis of relationship between predictors, and analysed endpoints to develop multivariate predictive model. ROC analysis was applied for the identification of optimal cut-offs of continuous variables and description of overall predictive power of developed multivariate models. Analyses were computed using SPSS 25.0.0.1 (IBM Corporation, Armonk, NY, USA, 2018).

Post-hoc power analysis was performed for the calculation of the minimal sample size. The power of the study to detect given sample size was 0.91 (two means *t*-test).

## 3. Results

### 3.1. Predictive Factors for Persistence of Glucose Intolerance Postpartum

Based on the results of postpartum oGTT *n* = 22 (9%) subjects exhibited any form of glucose intolerance up to 1 year after delivery. Comparison of basic anthropometric, clinical and biochemical data between groups with and without PGI showed significantly higher FPG and calculated AUC under the oGTT curve in mid-trimester, more frequent HbA1c above 42 mmol/mol, increased prevalence of obesity and hypothyroidism, more frequent positive family history of diabetes and higher incidence of smoking (recent smoker or stop-smoker) in PGI group, for details see Table 1 and Table 2. Intensive mode of insulin therapy (more than 3 times a day) was not predictive for PGI. 

**Figure 1:**
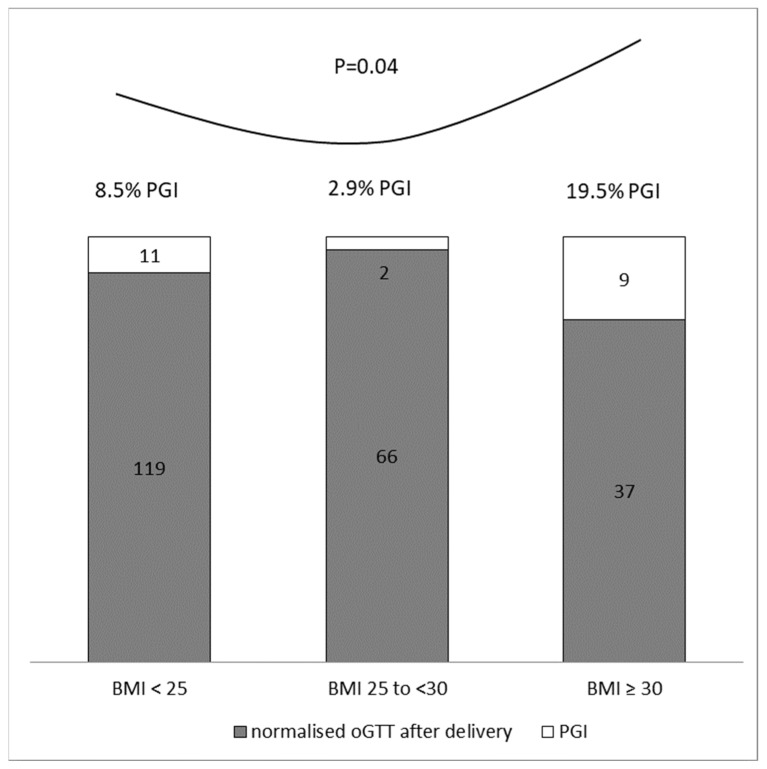
Comparison of postpartum PGI prevalence between the groups defined according to the preconception BMI. Numbers and percentages of GDM women with or without PGI. Risk of PGI is increased in the group with obesity (BMI ≥ 30) compared to the group with normal weight (BMI < 25), *p* = 0.04, chi-square test. Minimal risk of PGI is associated with overweight (i.e., BMI 25 to <30). BMI—body mass index; PGI—persisting glucose intolerance.

Subsequently, we stratified women with GDM according to their weight. Figure 1 shows a comparison of the incidence of PGI in the particular subgroups of women divided according to BMI (normal weight, BMI < 25; overweight, BMI 25 to <30; obesity, BMI ≥ 30). The risk of PGI was increased in obese women compared to normal weight (*p* = 0.04, chi-square test). Lowest risk of PGI seems to be in the group with an overweight. 

ROC analysis was performed with aim to find optimal cut-offs of particular monitored variables for prediction of persisted PGI. We identified 5.1 mmol/L as an optimal cut-off for FPG in mid-trimester of oGTT test (*p* < 0.001, 95% CI (0.698–0.810)) and 13.5 mmol/L as an optimal cut-off for AUC calculated from a 3-point oGTT in mid-trimester (*p* = 0.011, 95% CI (0.604–0.728)), all variables are shown in the Table 3.

### 3.2. Evaluation of Adverse Peripartal Outcomes

Data concerning delivery were available in 77% of women with GDM (*n* = 187), of those 169 had normal oGTT after delivery and 18 had PGI. Clinical, anthropometric and biochemical data, as well as comorbidities were similarly distributed in GDM with vs. without PGI in this subgroup compared to the whole group. Remaining 57 women finally chose to deliver in different hospitals and their peripartal data were not available. Evaluation of peripartal adverse outcomes revealed a significantly higher incidence of prolonged delivery in the PGI group. Furthermore, prevalence of caesarean sections or instrumental deliveries (i.e., using vacuum extractor or forceps) was also higher in the PGI group. On the contrary, the induction of delivery was associated with decreased the risk of PGI in our study group. All data are summarised in the Table 4.

### 3.3. Risk Stratification

**Figure 2:**
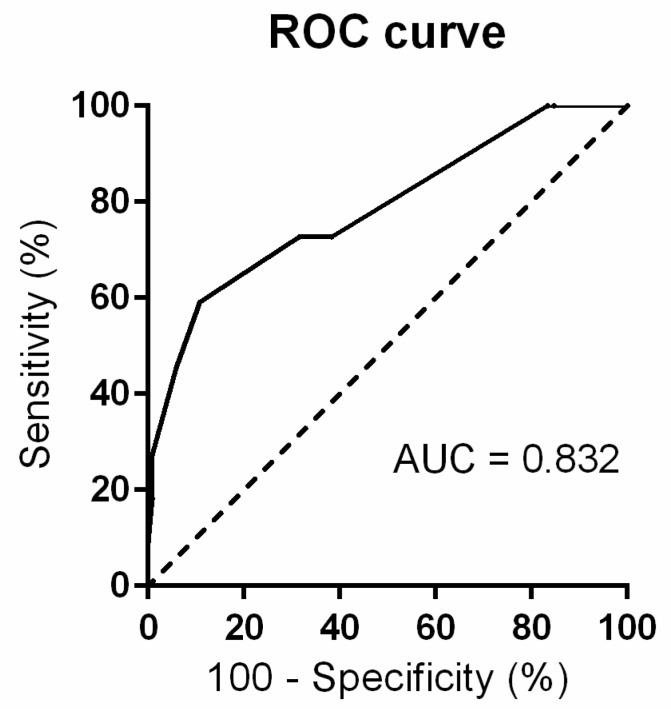
ROC curve for the “FOBDIT” score. The overall predictive accuracy of the risk score for PGI was 0.832 and the sensitivity and specificity were 0.824 and 0.903, respectively. Analysis was carried out using GraphPad Prism^®^ software, version 6.07.

Five parameters from the Table 2, Table 3 and Table 4 with highest significance and odds ratios (using backward stepwise likelihood-ratio method) were chosen to create a risk score for identification of women with a high risk for PGI development: (1) FPG in mid-trimester oGTT above 5.1 mmol/L (contributing by 2 points, OR 12.4, 95% CI (3.8–40.5), *p* < 0.001), (2) obesity (2 points, fixed parameter), (3) family history of diabetes (2 points, OR 5.8, 95% CI (0.7–48.2), *p* = 0.106), (4) instrumental delivery (2 points, OR 0.2, 95% CI (0.1–1), *p* = 0.049) and (5) personal history of hypothyroidism (1 point, OR 4.1, 95% CI (1.1–15.4), *p* = 0.034), with 9 points as a maximum. ROC analysis for a scoring model was conducted with the AUC 0.832, a highest specificity/sensitivity ratio (1.727) for 5 points as an optimal cut-off was calculated. Odds ratio (OR) for 5 and more points was 14.5 (*p* < 0.001, 95% CI (4.6–41.1)). Sensitivity was 0.824, specificity was 0.903. ROC curve is shown in Figure 2.

## 4. Discussion

Identification of risk factors involved during pregnancy might facilitate diagnostics and, also, early intervention of PGI and potentially lead to prevention of late complications related to GDM/T2DM. 

Our pilot study focusing on early postpartum conversion to PGI (up to 1 year after indexed delivery) identified several clinical and biochemical parameters that might be associated with increased risk of early development of PGI, specifically high FPG and HbA1c in the second trimester of pregnancy. Furthermore, we demonstrated that familiar history of diabetes, hypothyroidism, obesity and smoking are more common in women with postpartum PGI. All these parameters might point out to etiopathogenic heterogeneity of GDM.

Several studies focusing on similar topic were able to find relevant predictors of conversion of GDM to any type of DM after delivery ascertainable from the pre-pregnancy period. In a Swedish study, 174 women with GDM were followed up to 5 years postpartum [15]. The incidence of diabetes 5 years postpartum reached 30% in their cohort and major predictors of diabetes measured during pregnancy were HbA1c and FPG. HbA1c > 4.7% and FPG > 5.2 mmol/L during pregnancy were associated with four to six-fold increased risk of diabetes. Furthermore, family history of diabetes and the number of previous pregnancies increased the risk. Another study followed women with GDM, abnormal glucose tolerance and control healthy women for 6.75 years after delivery to determine the prevalence of DM, impaired glucose tolerance (IGT) and impaired fasting glucose and to identify predictors of DM postpartum [16]. They identified number of risk factors of diabetes in future life including family history of diabetes, FPG during pregnancy > 5.5 mmol/L, abnormal values of oGTT during pregnancy, age at delivery > 33 years, previous GDM and family history of diabetes. Abnormal glucose tolerance during pregnancy was associated with increased risk of diabetes postpartum although being lower than in women with GDM. In another study 487 women that underwent diagnostic oGTT in pregnancy and oGTT at 3 months after delivery were stratified into four groups of glucose tolerance based on results of glucose challenge test or oGTT [17]. The authors found that the risk of postpartum conversion rises with advancing degree of glucose intolerance (characterised by decrease of insulin sensitivity and β cell function). They also suggest that postpartum oGTT might be beneficial to detect those in high risk of conversion. Clinical parameters measured in the pregnancy in women with GDM were retrospectively analysed to find out whether they might predict abnormal glucose tolerance 6–12 weeks and 5.5 years postpartum [18]. The authors identified FPG > 5.4 mmol/L and 2-h glucose in oGTT > 9.3 mmol/L measured in pregnancy as a risk factor for glucose tolerance postpartum.

Several studies also focused on HbA1c as a possible predictor of PGI and their meta-analysis was performed [19]. The authors concluded that HbA1c itself is not sufficiently sensitive to predict postpartum transition of GDM to permanent diabetes. However, recent study reported that HbA1c values in the highest quartile (>36 mmol/mol) are associated with 5.5-fold higher risk of diabetes up to 5 years postpartum [20].

Fewer studies focused on possible predictors of peripartal outcomes in women with GDM. Large retrospective analysis showed that abnormal results of oGTT performed in pregnancy represented increased risk of preterm labour and admission to the neonatal intensive care unit [21]. Another study showed higher prevalence of large for gestational age (LGA) newborns in women with GDM than in healthy women (30.7% vs. 5.0%). The authors identified increased FPG and 1-h plasma glucose in oGTT as a risk factors for LGA [22]. 

To our knowledge, only few studies so far performed analysis of peripartal outcomes in relation to risk of postpartum PGI. In our study PGI group revealed a significantly higher incidence of prolonged deliveries and deliveries that need to be terminated by caesarean section or using instruments. The similar results were presented in an Australian study, which also confirmed our findings that women with PGI experienced significantly more often selected delivery complications such as the need for caesarean section or prolonged delivery [18]. Regardless the finding of the induction of delivery being associated with decreased risk of PGI in GDM women in our study, this parameter was not considered in the scoring system since overall frequency of labour induction in our study population was low and indications for the induction of delivery might vary among countries and as such might not be universally applicable. 

We perceive as very important to identify women at higher risk of PGI postpartum already during pregnancy and/or delivery since more than 50% of GDM women worldwide do not participate recommended postpartum re-screening (oGTT up to 6 months after delivery) [4,23,24]. Yet, attempts to quantify the risk (by calculating a risk score based on routine parameters) for developing diabetes postpartum in women with previous GDM are rather rare. We found only single published risk score according to Köhler et al. [25] that might be a bit complicated to calculate since it uses some parameters unavailable at the time of discharge (such as history of breast feeding on top of BMI, insulin in the therapy and family history of diabetes). We are therefore proposing our straightforward version of the PGI risk score denoted “*FOBDIT*” based on five routinely assessed parameters: “F” for fasting plasma glucose above 5.1 mmol/L in mid trimester oGTT, “OB” for obesity before pregnancy, “D” for diabetes in family history, “I” for instrumental delivery, “T” for hypothyroidism, with maximum of 9 points. Cut off value ≥ 5 points was associated with 14.5-times higher risk for PGI development after delivery in our group. 

Strength of the study lie in the fact that our proposed risk score identifying women with higher risk of PGI does not require any additional tests over the ones routinely performed and is therefore easily applicable in the clinical practice. Personalisation of the follow-up care can be performed at the moment when woman leaves a maternity hospital by means of simple calculation of the PGI risk and those at risk can be specifically motivated to attend the recommended oGTT test 6 weeks after delivery and booking can be performed immediately. Pending the manner of delivering this information and explaining the implications of PGI compliance can therefore be increased.

Dominant limitation of our study consists in a relatively small sample of participants, especially in PGI group and loss of nearly a quarter of the peripartal data in women who chose to deliver outside the university hospital. Furthermore, since our study population was ethnically homogenous and included solely Caucasian subjects, replication on a larger independent population comprising also non-Caucasian ethnicities is desirable should the “*FOBDIT*” risk score be used as a clinically validated tool.

## 5. Conclusions

We confirmed the results of our earlier study [26] (in a different cohort of women, but with the same diagnostic criteria) focused on incidence of GDM conversion into persistent impaired glucose tolerance: (i) the prevalence of PGI up to 1 year after delivery repeatedly reached about 10% (in previous study 11.7%, vs. now 9%), and (ii) higher FPG in oGTT test and a higher HbA1c in the second trimester seem to be very relevant predictors of persistent PGI. Moreover, recent study identified other potentially useful parameters (obesity, diabetes in family history, hypothyroidism and smoking) what could become a guide to make decision about a possible enhanced stratification of pregnant women with GDM and potential stronger recommendation to postpartum monitoring of risk groups. 

We suggest a new risk score called “*FOBDIT*” by using simple parameters identified before and during delivery which could be a useful tool for diabetologists and obstetricians with a possibility to stratify the population of women with GDM and to focus on women with higher postpartum risk of developing diabetes after delivery. It seems that delivery induction may play an important role in further life of GDM women but there is a strong need to validate the findings in larger cohorts.

## Figures and Tables

**Table 1:** Clinical, anthropometric and biochemical data of GDM women with and without PGI.

Parameter	Normalised oGTT after Delivery (*n* = 222)	Prediabetes/Diabetes after Delivery (*n* = 22)	*p*
Age (years)	32 (30–35)	32 (28–34)	NS
Primiparity	45%	36.4%	NS
Child birth weight (g)	3185 (2920–3500)	3360 (3030–3630)	NS
Insulin (intensive mode)	36.5% (79%)	45.5% (90%)	NS
Pre-gestational BMI (kg/m^2^)	24.7 (21.7–28.3)	24 (20.4–32.7)	NS
Weight increment up to mid trimester (kg)	6.8 (4–9)	6.5 (4–8)	NS
Total weight increment during pregnancy (kg)	8 (5.8–10)	9 (6–11)	NS
Systolic blood pressure in mid trimester (mmHg)	114 (105–125)	124 (112–128)	NS
Diastolic blood pressure in mid trimester (mmHg)	74 (68–81)	79 (69–87)	NS
HbA1c (mmol/mol)	33 (31–36)	35 (32–36)	NS
HbA1c > 42 mmol/mol	1.8%	9.1%	0.035
oGTT fasting plasma glucose in mid trimester (mmol/L)	4.7 (4.4–5.0)	5.3 (4.8–6.1)	<0.001
oGTT 1-h post-75 g load in mid trimester (mmol/L)	9.4 (9.0–10.0)	8.3 (8.2–8.8)	NS
oGTT 2-h post-75 g load in md trimester (mmol/L)	8.2 (7.7–8.9)	8.1 (7.8–9.2)	NS
AUC calculated from a 3-point oGTT in mid-trimester	13.0 (12.3–13.6)	13.6 (12.8–14.6)	0.025
oGTT fasting plasma glucose postpartum (mmol/L)	4.6 (4.4–4.9)	5.7 (5.1–5.8)	<0.001
oGTT 1-h post-75 g load postpartum (mmol/L)	6.7 (5.4–7.9)	7.9 (6.7–10.1)	0.048
oGTT 2-h post-75 g load postpartum (mmol/L)	5.0 (4.3–5.8)	7.7 (6.1–8.3)	<0.001

Data expressed as a median (IQR) or proportions. Differences evaluated by nonparametric Mann–Whitney or chi-square test, respectively. GDM—gestational diabetes mellitus; PGI—postpartum glucose intolerance; oGTT—oral glucose tolerance test; BMI—body mass index; HbA1c—glycated haemoglobin; AUC—area under oGTT curve.

**Table 2:** Comorbidities in GDM women with and without PGI.

Parameter	Normalised oGTT after Delivery (*n* = 222)	Prediabetes/Diabetes after Delivery (*n* = 22)	*p*
Diabetes mellitus in family anamnesis	68.9%	95.5%	0.0087
Smoker or stop-smoker	17.6%	36.4%	0.033
Obesity (BMI ≥ 30 kg/m^2^)	17.1%	40.9%	0.007
Hypothyroidism *	13.1%	31.8%	0.018
Preeclampsia/hypertension	9%	18.2%	NS
Polycystic ovary syndrome *	1.4%	0%	NS
Thrombophilia *	5.4%	9.1%	NS
Anaemia *	14.9%	13.6%	NS
Allergy *	35.1%	31.8%	NS
Polymorbidity (≥3 illnesses incl. GDM)	32.9%	63.6%	0.004

Data expressed as proportions. Differences evaluated by chi-square test, respectively. GDM—gestational diabetes mellitus; PGI—postpartum glucose intolerance; oGTT—oral glucose tolerance test; BMI—body mass index. * Data about comorbidities were obtained from anamnesis or according to an established medication.

**Table 3:** ROC analysis: optimal cut-offs of selected glycaemic indices for prediction of postpartum GDM conversion into permanent glucose abnormality.

Parameter	Cut off According to Youden Index ^a^	AUC_ROC_	95% CI ^b^	*p*
oGTT fasting plasma glucose in mid trimester (mmol/L)	>5.1	0.757	0.698–0.810	< 0.001
oGTT 1-h post-75 g load in mid trimester (mmol/L)	>8.8	0.719	0.606–0.814	0.231
oGTT 2-h post-75 g load in mid trimester (mmol/L)	>7.6	0.570	0.504–0.634	0.361
HbA1c (mmol/mol)	>33.0	0.602	0.538–0.664	0.087
Pre-gestational BMI (kg/m^2^)	>28.9	0.502	0.437–0.566	0.985
AUC calculated from a 3-point oGTT in mid-trimester	>13.5	0.668	0.604–0.728	0.011

^a^ Youden index (Youden, 1950) (i.e. sensitivity+ specificity-1), ^b^ Binomial exact (binomic exact test for the calculation of confident intervals (CI)). ROC—Receiver Operating Characteristic; GDM—gestational diabetes mellitus; AUC_ROC_—area under the ROC curve; CI—confidence interval; oGTT—oral glucose tolerance test; HbA1c—glycated haemoglobin; BMI—body mass index; AUC—area under the curve.

**Table 4:** Comparison of peripartal adverse outcomes in women with GDM with and without PGI.

Parameter	Normalised oGTT after Delivery (*n* = 169)	Prediabetes/Diabetes after Delivery (*n* = 18)	*p*
Macrosomia (child birth weight above 4000 g)	15.4%	22.2%	NS
Pre-term delivery (before 38th week of gestation)	8.9%	11.1%	NS
Delivery induction (using oxytocin or prostaglandin E)	41.4%	16.7%	0.037
Non-physiologic delivery (caesarean section, VEX using, forceps using)	25.4%	55.6%	0.007
Prolonged delivery (=above 480 min)	18.3%	38.9%	0.04
Complications after delivery (manual extraction of placenta, hypotonia uteri)	5.9%	5.6%	NS
Abnormal Apgar score (in 5th min <5)	1.8%	5.6%	NS
Abnormal cord blood pH (<7.1)	0.6%	5.6%	NS
Abnormal BE (<−12)	1.2%	5.6%	NS

Comparison was performed using chi-square test. GDM—gestational diabetes mellitus; PGI—postpartum glucose intolerance; oGTT—oral glucose tolerance test; VEX—vacuum extractor; BE—base excess.

## Data Availability

On request by authors.
